# Therapeutic Development by Targeting the cGAS-STING Pathway in Autoimmune Disease and Cancer

**DOI:** 10.3389/fphar.2021.779425

**Published:** 2021-11-15

**Authors:** Qiumei Li, Shuoran Tian, Jiadi Liang, Jiqiang Fan, Junzhong Lai, Qi Chen

**Affiliations:** ^1^ Fujian Key Laboratory of Innate Immune Biology, Biomedical Research Center of South China, Fujian Normal University, Fuzhou, China; ^2^ The Cancer Center, Union Hospital, Fujian Medical University, Fuzhou, China

**Keywords:** innate immunity, cGAS-STING pathway, autoimmune disease, cancer, therapeutic development

## Abstract

DNA immune recognition regulation mediated by the cGAS-STING pathway plays an important role in immune functions. Under normal physiological conditions, cGAS can recognize and bind to invading pathogen DNA and activate the innate immune response. On the other hand, abnormal activation of cGAS or STING is closely related to autoimmune diseases. In addition, activation of STING proteins as a bridge connecting innate immunity and adaptive immunity can effectively restrain tumor growth. Therefore, targeting the cGAS-STING pathway can alleviate autoimmune symptoms and be a potential drug target for treating cancer. This article summarizes the current progress on cGAS-STING pathway modulators and lays the foundation for further investigating therapeutic development in autoimmune diseases and tumors.

## Introduction

### Innate Immunity and Immune Diseases

#### Innate Immunity and cGAS-STING Signaling Pathway

**FIGURE 1:**
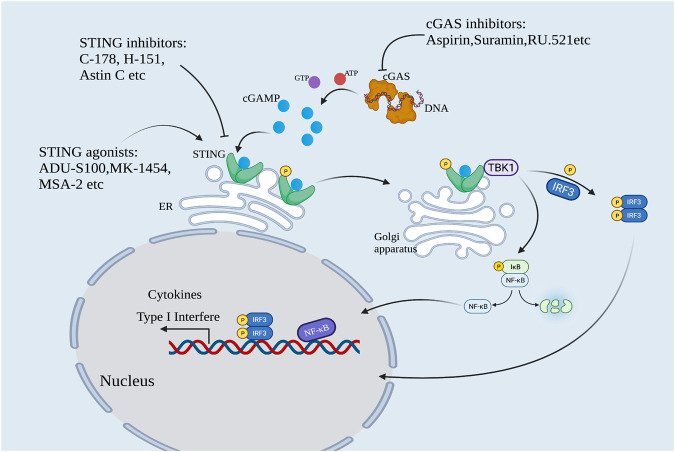
The cGAS-STING pathway. cGAS is activated by sensing cytoplasmic DNA. Activated cGAS catalyzes the formation of cGAMP. cGAMP activates STING protein, and activated STING recruits TBK1 and phosphorylates IRF3. Meanwhile, STING activated IKK kinase and phosphorylated IκB, leading to the release of NF-κB. Phosphorylated IRF3 formed dimer and translocated with NF-κB into the nucleus to synergistically induce the expression of IFN-I and various inflammatory factors. This pathway can be regulated by cGAS and STING modulators.

The human immune system uses pattern recognition to sense infection and trigger an immune response against pathogen invasion. At the beginning of the 21st century, TLR9 was the only known foreign DNA pattern recognition receptor (PRR). In 2006, Medzhitov and Stetson reported a novel DNA-sensing immune response independent of TLR9 which can lead to interferon regulatory factor 3 (IRF3) mediated type I interferon production (Stetson and Medzhitov, 2006). They further found that the abnormal accumulation of cytoplasmic DNA caused by the abnormality of 3′ repair exonuclease 1 (Trex1) could be sensed by unknown DNA receptors leading to fatal autoimmune symptoms (Stetson et al., 2008). By the end of 2012, Chen’s team discovered a novel second messenger molecule, guanine cyclic dinucleotide (cGAMP), and its synthase cGAMP synthase (cGAS), and demonstrated that cGAS could recognize abnormal DNA in the cytoplasm and induce an innate immune response named “the cGAS-STING signaling pathway” (Ablasser et al., 2013). In the absence of double-stranded DNA (dsDNA), cGAS is in a dormant state. When a virus invades the body or when cell damage causes abnormal dsDNA accumulation in the cytoplasm, cGAS can recognize and bind to dsDNA, actively form a dimer, and catalyze the synthesis of ATP and GTP into cGAMP with phosphodiester bonds (2′3′-cGAMP). cGAMP is an endogenous ligand of STING protein located on the endoplasmic reticulum membrane. After STING is activated, the conformation of STING changes and STING moves from the endoplasmic reticulum to the Golgi apparatus, and then recruits TANK-binding kinase 1 (TBK1) and phosphorylates IRF3. Phosphorylated IRF3 forms a dimer and enters the nucleus. At the same time, STING can also activate IKK kinase (inhibitor of nuclear factor kappa-B kinase) and phosphorylate IκB, causing its degradation and the release of NF-κB. IRF3, NF-κB, and other regulatory factors in the nucleus work together to induce the expression of type I interferon (IFN-I) and various inflammatory factors (e.g., TNF, IL-6, and IL-1β), and initiate the innate immune response (Figure 1).

#### Autoimmune Diseases and the cGAS-STING Signaling Pathway

The cGAS-STING pathway plays an important role in innate immunity, but cGAS can also be activated by the body’s abnormal DNA to cause tissue damage or autoimmune diseases, such as Aicardi-Goutières syndrome (AGS), systemic lupus erythematosus (SLE), primary biliary liver disease. Genetic studies have shown that mutations in the genes prevent abnormal accumulation of cytoplasmic DNA, such as DNA exonuclease Trex1 (Crow et al., 2006), deoxyribonuclease-II (DNAse-II) (Kawane et al., 2001), and adenosine deaminase ADAR1 (Hartner et al., 2009), can lead to these diseases. Our group constructed a Trex1-D18N point mutation model in mice by using the CRISPR/Cas9 technology. The mice exhibit a systemic inflammatory phenotype, similar to familial chilblain lupus (FCL) and SLE. In this model, the inactivation of TREX1 leads to abnormal accumulation of dsDNA in the cytoplasm, which leads to the overexpression of IFN-I. However, after the cGAS gene is knocked out, abnormal IFN-I levels return to normal, and systemic inflammatory response and abnormal activation of T cells are effectively alleviated (Xiao et al., 2019), underscoring the role of the cGAS-STING pathway in such diseases and making it an important target for the development of drugs to treat these diseases (Abe et al., 2013).

On the other hand, the cGAS-STING pathway is also an important monitoring mechanism in the body’s antitumor immunity. In the process of immune surveillance, cGAS can detect the DNA leaked into the cytoplasm during abnormal mitosis that often occurs in malignant cells, induce the secretion of IFN-I, which stimulates the presentation of tumor antigens, and activates tumor-specific CD8^+^ effector T cells to exert the antitumor effect (Duewell et al., 2014). In addition, dendritic cells (DCs) can recognize tumor-derived DNA to express IFN-I and initiate antitumor immunity (Deng et al., 2014). It is worth noting that the cGAS-STING pathway is defective in many cancer types, including melanoma and colorectal cancer (Ridker et al., 2017). Increasing evidence indicates that the specific activation of STING can stimulate innate immunity and promotes T cell infiltration into the tumor microenvironment (TME), thereby suppress tumor progression (Düwell et al., 2019).

Modulating the cGAS-STING pathway and expression of IFN-I and related inflammatory factors are important in alleviating autoimmune diseases caused by immune abnormalities. In addition, cGAS and STING can serve as a bridge connecting innate immunity and adaptive immunity and regulate the occurrence and development of malignant tumors (Parlato and Yeretssian, 2014). Therefore, targeting the cGAS-STING pathway has great therapeutic potential and is receiving much attention in the pharmaceutical field. In the following, we summarized the current progress in developing molecular agents targeting the cGAS-STING pathway, and their therapeutic potential is also discussed.

## cGAS Inhibitors

### Structural Features of cGAS

**FIGURE 2:**
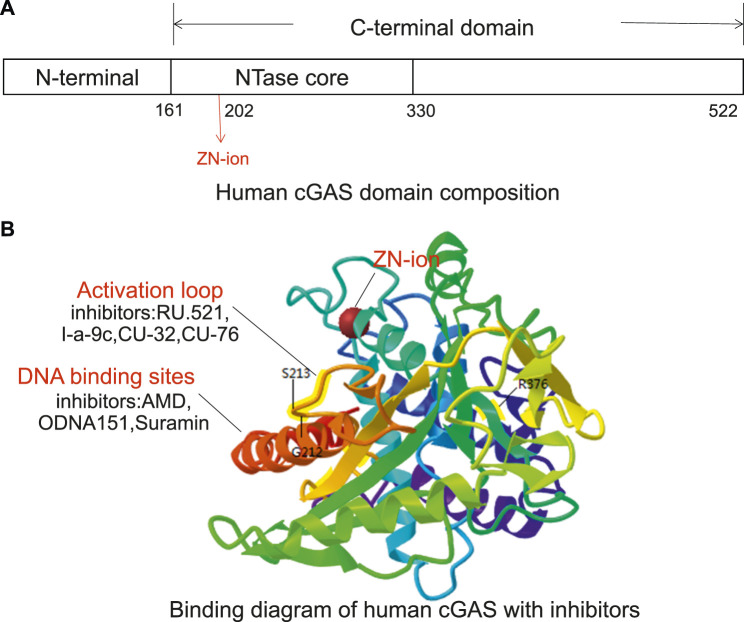
The potential targeting sites of cGAS modulators, as shown on the X-ray crystal structure of human cGAS (Adopted from PDB ID: 4KM5). **(A)** Human cGAS domain composition. The human cGAS structure contains N-terminal helical extensions (amino acid residues 1–160) and C-terminal domains (amino acid residues 161–522). A unique zinc ion is located at position 202. **(B)** Binding diagram of human cGAS with inhibitors. The G212, S213, and R376 sites in the activation loop are the key sites of cGAS binging substrate (ATP + GTP). The inhibitors that target DNA binding sites include AMD, ODNA151, and suramin, and the inhibitors targeting ATP and GTP binding sites include RU.521, CU-32, CU-76, and I-a-9c.

The cGAS protein is a member of the nucleotide transferase family. It contains an N-terminal domain and a C-terminal domain with a specific zinc-ion-binding module (Figure 2). The zinc-band structure mediates the binding of cGAS to the phosphate ribose backbone of dsDNA and the dimerization of cGAS and participates in the synthesis of 2′3′-cGAMP. The catalytic domain of cGAS is the NTase family, whose N-terminus contains all catalytic residues (Zhang et al., 2020). In 2013, Kranzusch et al. (2013), Kato et al. (2013), and Li et al. (2013a) reported the crystal structure of cGAS protein without binding dsDNA or ligand. Point mutation studies have shown that certain amino acid residues such as Lys335 (m-cGAS)/Lys347 (h-cGAS) are important for forming cGAS dimers and cGAS functions. cGAS activity is eliminated by Lys335 and Lys382 (m-cGAS)/Lys394 (h-cGAS) point mutations (Zhang et al., 2014). In addition, Tyr436 and Arg376 can form π-π stacking and π-cation with the aromatic center of the ligand, respectively. The amino group of Lys362 can form a salt bridge with the phosphate group of cGAMP, and the amino hydrogen on Asp227 adenine can interact with Asp319. The carboxyl oxygen forms a hydrogen bond. These amino acids exert a synergistic effect in cGAS functions (Chen, 2019). The crystal structure analysis provides a foundation for structure-based drug design and development (Hall et al., 2017a).

### Current cGAS Inhibitors

The development of cGAS inhibitors is at its initial stage. The currently known cGAS inhibitors can be divided into non-substrate competitive inhibitors and substrate competitive inhibitors according to whether they act on the active site of cGAS substrates. Non-substrate competitive inhibitors usually inhibit the activity of cGAS by binding to the necessary groups other than the active center, such as aspirin. Substrate competitive inhibitors usually share structural similarities with the substrate for binding, thereby reversibly inhibiting enzyme activity, such as RU.521. The following is a detailed description of the currently known cGAS inhibitors (Table 1).

**TABLE 1:** cGAS inhibitors.

cGAS inhibitors	Inhibition mechanism	Biological effect	Refs
Aspirin	Acetylated Lys amino group of cGAS protein	Improved DNA-mediated autoimmune responses in mouse and AGS patient cells	Shakespear et al. (2011); Dai et al. (2019)
AMDs	It binds to dsDNA and occupies the binding site of cGAS	In THP-1 cells, the IC_50_ dose range of AMDS was 3–25 μM	An et al. (2015); Bose et al. (2016)
ODN A151	Telomere sequences and thiophosphate mainchains compete with DNA for cGAS	Inhibit the expression of type I interferon in THP-1 cells and the activation of cGAS in its Trex1^−/−^ cells	Steinhagen et al. (2018)
RU.521	Occupy the catalytic sites of cGAS and competes with ATP	The IC_50_ in macrophages is 700 nM	Vincent et al. (2017); Lama et al. (2019)
PF-06928215	Binding to cGAS active site	It was verified in THP-1 cells	Hall et al. (2017b); Zhao et al. (2020)
Suramin	As a nucleic acid analog, it prevents cGAS from binding to dsDNA	Regulates the production of IFN-I in THP-1 cells	Wang et al. (2018); Padilla-Salinas et al. (2020)
I-a-9c	At the DNA binding site of cGAS, Tyr436, Arg376, and Asp227 form forces	The inhibition rate of cGAS was 83.9% at the 10 μM level	Chen (2019)
CU-32	The zinc capsule structure inserted into cGAS inhibits the formation of dimer	The cGAS-STING pathway was specifically inhibited	Hall et al. (2017b); Padilla-Salinas et al. (2020)
CU-76			

#### Aspirin

The classic drug aspirin is a non-steroidal anti-inflammatory drug (NSAID), which is known to acetylate proteins such as cyclooxygenase (COX) (Shakespear et al., 2011). Studies have shown that aspirin can inhibit the activity of human cGAS by regulating its post-translational modification in patient cells by acetylating Lys384, Lys394, or Lys494 (Dai et al., 2019). Aspirin improves DNA-mediated autoimmune responses in mice and patients with AGS by inhibiting the function of cGAS (Dai et al., 2019). At present, aspirin is widely used in clinical practice, with 2,269 items registered clinical trials on the NIH list. Its pharmacological action and pharmacological metabolism have been well defined. These findings suggest that aspirin can act as a human cGAS inhibitor for the treatment of immune diseases.

#### Antimalarial Drugs

Antimalarial drugs (AMDs), a family of widely used drugs for the treatment of malaria, have proved a safety profile. In 2015, An et al. reported a series of AMDs that can interfere with the binding of cGAS and dsDNA, including hydroxychloroquine sulfate (HCQ), chloroquine (CQ), and quinine (QN) (An et al., 2015). The results show that HCQ can inhibit cGAS activity by non-specific binding of aminoquinoline and aminoacridine, taking up the DNA binding sites R342 and K372. In addition, their inhibition of cGAS activity and IFN-β production is dose-dependent. For example, the half-maximal inhibitory concentration (IC_50_) of AMDs in THP-1 cells is in the dose range of 3–25 μM, while the IC_50_ of QN is 13 μM (Bose et al., 2016). Because of the good safety profile of AMDs and its inhibitory capability on cGAS, the interaction between AMD and cGAS provides a new therapeutic strategy for the treatment of innate immune diseases.

#### An Oligodeoxynucleotide Containing a TTAGGG Modified Fragment (ODNs)

In 2018, Steinhagen et al. reported that ODNs containing the TTAGGG modified fragment could inhibit cGAS activity (Steinhagen et al., 2018). It inhibited the expression of type I interferon in THP-1 cells and the activation of cGAS in *Trex1* ^*−/−*^ cells. Among them, the inhibitory activity of ODN A151 depends on the telomere sequence and phosphorothioate backbone to prevent cGAS activation by competing with DNA (Steinhagen et al., 2018), laying the foundation for developing new immunosuppressive agents to treat autoimmune diseases caused by cGAS abnormal activation.

#### The RU Series of Compounds

Some drugs bind to cGAS, thereby affecting the affinity of ATP or GTP to cGAS, which is the key to inhibition. In 2017, Vincent et al. reported that the RU series of compounds could occupy the catalytic sites Arg364 and Tyr421 of cGAS in mice, reduce the binding affinity of cGAS to ATP and GTP suppress the expression of interferon in primary macrophages (Vincent et al., 2017). RU.521 showed good activity in the macrophages derived from the AGS mouse model (IC_50_ = 700 nM). Due to the low signal of human cGAS (h-cGAS) in RF mass spectrometry, accurate kinetic characterization cannot be carried out (Lama et al., 2019). Based on the significant inhibition of the RU series of compounds on murine cGAS, the RU series of compounds are expected to be used as human cGAS inhibitors but need further investigation.

#### The PF Series of Compounds

In 2017, Hall et al. reported the PF series of compounds with a high affinity for binding human cGAS by a novel fluorescence polarization method (Hall et al., 2017b). The study found that PF-06928215 bound to cGAS efficiently and showed high inhibitory activity *in vitro*. Later, Zhao’s research group reported the discovery of a novel human cGAS catalytic domain (H-cGAS^CD^) and screened out the PF compounds S2 (IC_50_ = 13.1 ± 0.09 μM) and S3 (IC_50_ = 4.9 ± 0.26 μM) as h-cGAS inhibitors (Zhao et al., 2020). These studies lay a foundation for the further application of PF compounds.

#### Suramin

Suramin has a variety of functions and many clinical applications. So far, there are 21 suramin-related clinical trials on the NIH list. Its toxicological characteristics and target structure are clear. In 2018, Wang et al. reported that suramin could inhibit cGAS and regulate the production of type I interferon (Wang et al., 2018). It is showed that suramin, as a nucleic acid analog, blocks the binding of cGAS to dsDNA. However, suramin may interact with the Toll-like receptor 3 (TLR 3) pathway to produce off-target effects as well (Padilla-Salinas et al., 2020). Therefore, structure optimization of suramin needs to be further conducted.

#### Substituted Acetamides

At present, few skeleton structures of cGAS nucleoside site inhibitors have been reported. In 2019, Chen reported the synthesis of cGAS inhibitor pharmacophore based on the cGAS receptor-ligand complex structure (Chen, 2019). Conformational analysis shows that the original receptor-ligand binding effect between the compound I-a-9-c and cGAS. Tyr436 and Arg376 can form π-π stacking and π-cation with the aromatic center of the I-a-9c, respectively. In addition, the hydroxyl group on its propanol group can also form a hydrogen bond with the carboxyl group of Asp 227 in cGAS, and the hydrogen bond improves the inhibition of cGAS activity. The inhibitory rate of I-a-9c on the cGAS activity at 10 μM is 83.9% in THP-1 cells (Chen, 2019). These compounds have low toxicity and high efficiency, so it has potential for further development.

#### The CU Series of Compounds

Crystal structure studies have shown that the two DNA-binding surfaces and the protein-protein interface (PPI) of cGAS play an important role in IRF3 activation and IFN-β induction (Hall et al., 2017b). In 2019, Padilla-Salinas et al. reported a novel drug binding site of cGAS at Z9189 by targeting the PPI of human cGAS (Padilla-Salinas et al., 2020). Structural docking indicated that the inhibitor CU series of compounds targeting Z9189 might be inserted into the zinc capsule structure of cGAS, thus inhibiting dimer formation through the allosteric effect. It is worth noting that CU-32 and CU-76 specifically inhibit the cGAS-STING pathway but have no significant effect on the RIG-I-MAVs pathway or the TLR pathway. Further studies showed that the IC_50_ values of CU-32 and CU-76 in THP-1 cells were 0.66 and 0.27 μM, respectively, and the inhibitory effect was dose-dependent (Padilla-Salinas et al., 2020), which provides a new chemical scaffold for developing cGAS inhibitors.

## Research Status of STING Modulators

Increasing evidence indicates that persistent activation of STING is associated with the pathogenesis of autoimmune diseases induced by its gene mutations, such as AGS (Barber, 2015), autoimmune myocarditis (Kwon and Bakhoum, 2020), multiple arthritis, and other related vascular diseases (Feng et al., 2020). These diseases occur in infants with family history and pose a serious threat to human life and health. Therefore, STING is an attractive target for therapeutic intervention.

### Structural Characteristics of STING

**FIGURE 3:**
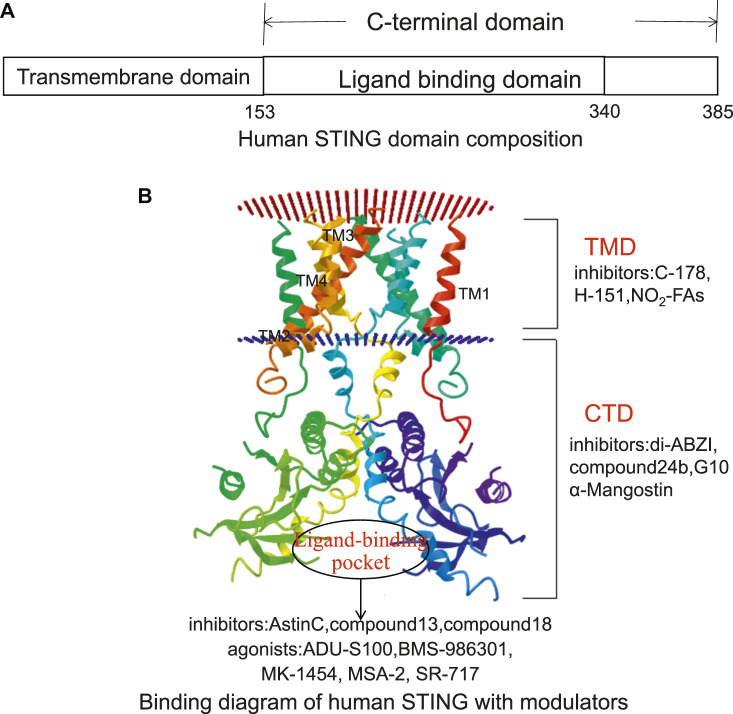
The potential targeting sites of STING modulators, as shown on the Cryo-EM structure of human STING (Adopted from PDB ID: 6NT5). **(A)** Human STING domain composition. Human STING protein, as a homologous dimer, is composed of a luminal N-terminal domain (TMD, amino acid residues 1–152) and a cytoplasmic C-terminal domain (CTD, amino acid residues 153–385) containing ligand-binding domain (amino acid residues 153–340). **(B)** Binding diagram of human STING with modulators. The inhibitors targeting the TMD domain include C-178, H-151, and NO_2_-FAs. The inhibitors targeting the CTD domain include di-ABZI, Compound 24b, G10, and α-Mangostin. The inhibitors targeting the ligand-binding sites include astin C, Compound13, and Compound18. Its agonists include ADU-S100, BMS-986301, MK-1454, MSA-2, and SR-717.

The human STING (h-STING) protein, as a homodimer, consists of a luminal N-terminal domain (four transmembranes helically anchored ER: TM1-4) and a cytoplasmic C-terminal domain (CTD) containing ligand binding pockets (Wang et al., 2014) (Figure 3). The crystal structure of CTD dimer shows that without cGAMP, the ligand-binding domain (LBD) is in an inactive open conformation. cGAMP binding induces the CTD of the STING dimer to turn clockwise relative to the transmembrane domain (TMD) (Wang et al., 2014). Rotating 180° causes the formation of β-sheets, covering the ligand-binding pocket, causing STING to become an active closed state. Point mutation studies have shown that the residues in the N-terminal helical loop, V147 L, N154 S, and V155 M, may contribute to the conformational change of STING. After the ligand binds to STING, the STING TMD is modified post-translationally, and STING was translocated from the endoplasmic reticulum to the Golgi apparatus, in which palmitoylation of Cys88/91 is crucial to the activation of STING. STING binds to the TBK1 dimer through the C-terminal, activates TBK1, and phosphorylates IRF3, and the main phosphorylation site is Ser366 at the C-terminal (Weerapana et al., 2010). The phosphorylation at Ser172 of TBK1 is required for its activation (Mukai et al., 2016). Although the specific mechanism of STING activation needs further investigation, these analyses lay the foundation for structure-based drug design to facilitate the research and development of novel immune regulatory agents with high efficiency and low toxicity. The currently known STING inhibitors are listed on Table 2.

**TABLE 2:** STING inhibitors.

STING inhibitors	Inhibition mechanism	Biological effect	Refs
C-178, H-151	It forms a covalent bond with TMD Cys91 and Cys88	The therapeutic effect was shown in the Trex1^−/−^ mouse tumor model	Haag et al. (2018)
NO_2_-FAs	It forms covalent bonds with Cys88/91 and N-terminal His16	It was demonstrated in fibroblasts from SAVI patients	Hansen et al. (2018)
Astin C	Binding to Ser162, Tyr163, and Arg238 occupy site pockets	The IC_50_ values in mouse and human fibroblasts were 3.4 and 10.8 μM, respectively	Burdette et al. (2011); Li et al. (2018)
Compound 18	Hydrogen bonding is formed with Thr263 by carboxyl group	EC_50_ = 30 μM, IC_50_ = 11 μM	Siu et al. (2019)

### STING Inhibitors

#### Nitrofuran Derivatives

Palmitoylation of STING is a post-translational modification critical for the assembly of STING into polymer complexes in the Golgi apparatus and recruitment of downstream signal factors (Zhang et al., 2013). In 2018, Haag et al. reported that nitrofuran derivative C-178 and indoles derivative H-151-Al (H-151) were irreversible inhibitors of mouse STING (m-STING) and human STING (h-STING), respectively (Haag et al., 2018). The main inhibitory mechanism was that C-178 forms covalent bonds with Cys91 and Cys88 of STING TMD, which affects the palmitoylation of STING. Unlike C-178 and H-151, Hansen et al. reported that nitro-fatty acids (NO_2_-FAs/CXA-10) had an inhibitory effect on either mouse or human STING (Hansen et al., 2018). NO_2_-FAs forms a covalent bond with Cys88/91 and N-terminal His16, which affects the palmitoylation of STING and inhibits TBK1 phosphorylation in the fibroblasts derived from patients of STING-associated vascular disease (SAVI). Additionally, CXA-10, a STING inhibitor, has also entered clinical trials as an oral peroxisome proliferator-activated receptor agonist (PPAR) for the treatment of primary focal segmental glomerulosclerosis (FSGS) (Hansen et al., 2018).

#### Astin C

STING can be directly associated with cyclic dinucleotides (CDNs) to activate the downstream immune response (Burdette et al., 2011). In 2018, Li et al. reported that astin C, a natural cyclic peptide from Aster, inhibited the innate immune CDN sensor STING (Li et al., 2018). Astin C specifically binds to the CTD region of STING and occupies the cGAMP binding pocket by interacting with Ser162, Tyr163, and Arg238 to inhibit h-STING functions. In isothermal titration calorimetry, astin C binding to STING can be abolished by high concentrations of cGAMP. In addition, astin C inhibited IFN-β mRNA expression in mouse and human fibroblasts with the IC_50_ values of 3.42 and 10.83 μM, respectively (Li et al., 2018). Based on the high efficiency and low toxicity. Astin C may be used to treat STING dysfunction-mediated diseases.

#### Derivatives Containing Carboxylic Acids

Targeting the large protein pocket in STING is a challenge since the molecular weight of its endogenous ligand cGAMP is relatively high (Burdette and Vance, 2013). In 2019, Siu et al. reported that by using the symmetry of STING protein, small molecules (derivatives containing carboxylic acids) were screened to bind to the open conformation of STING in the ratio of 2:1 (Siu et al., 2019). Such binding stoichiometry can fully occupy the large binding site while maintaining oral drugs’ good physical and chemical properties. As the antagonists of h-STING, the selected carboxylic acid derivatives of Compound 13 (Siu et al., 2019) (EC_50_ = 30 μM, IC_50_ = 11.5 μM) and 18 (EC_50_ = 30 μM, IC_50_ = 11 μM) formed hydrogen bonds with Thr263 through carboxyl groups and stabilized the open conformation of STING. With a binding ratio of 2:1, there is a risk of instability in the drug potency. Therefore, two-dimensional optimization of protein-ligand and ligand-ligand interactions is needed to improve valence efficiency (Siu et al., 2019).

### STING Agonists

Activating the cGAS-STING pathway can enhance the immune response and restrain tumor growth. In addition, STING agonists can be used as adjuvants to develop vaccines against certain infectious diseases, such as HIV and malaria. Currently, most STING activators are synthetic CDNs. The entry of cGAMP into cells can overcome the escape of cGAS recognition by pathogens (Li et al., 2013b), and activate the interferon response driven by STING in DCs, thereby promoting the formation of major histocompatibility complex presenting tumor-associated antigens to activate CD8^+^ T cells for antitumor killing (Li et al., 2016). Several known STING agonists are described below (Table 3).

**TABLE 3:** STING agonists.

STING agonists	Activation mechanism	Biological effect	Refs
ADU-S100, BMS-986301, MK-1454	Binding with LBD in STING	They are indicated for the treatment of advanced solid tumors with monotherapy and combined ICIs	Dubensky et al. (2013); Corrales et al. (2015); Meric-Bernstam et al. (2019) Ding et al. (2020) Harrington et al. (2018)
Di-ABZI, Compound 24b	Binding with Ser241 and Ser162	EC_50_ of di-ABZI was 130 nM; Compound 24b EC_50_ = 0.287 μM	Ramanjulu et al. (2018)
			Xi et al. (2020)
C11, BNBC	Binding with h-STING	Specifically activate STING mediated immune responses and effectively block replication of multiple alphavirus types	Gall et al. (2018)
			Zhang et al. (2019)
Kitacinnamycins 8	Binding with STING	Increased poly (dA:dT) and cGAMP-induced IFN-β expression	Shi et al. (2019)
DMXAA	Binding with m-STING	Activates the TBK1-IRF3 pathway and shows good activity in mouse solid tumors	Kim et al. (2013a)
α-Mangostin, G10	Binding with the CTD region of h-STING	Activated the STING-TBK1-IRF3 pathway. The EC_50_ of G10 ranged from 2.5–4.3 μM. α -Mangosteen can repolarize M2 macrophages into M1 phenotype	Zhang et al. (2018)
			Banerjee et al. (2020)
DSDP	Binding with h-STING	Induces STING-dependent cytokine responses in HFF and PBMCs cells and effectively inhibits the replication of a variety of viruses	Liu et al. (2017)
MSA-2	Binding with STING	Persistent antitumor immunity and synergistic anti-PD-1 therapy	Pan et al. (2020)
SR-717	Binding with STING	Antitumor activity	Chin et al. (2020)

#### Cyclic Dinucleotides

In recent years, CDNs have become a class of STING agonists with anticancer effects (Dubensky et al., 2013). In 2015, Corrales et al. reported that CDNs can bind to the ligand-binding domain of STING and activate it, thereby affecting the vascular system and tumor microenvironment and initiating the activities of APC (antigen-presenting cells) and CD8^+^ T cells (Corrales et al., 2015). Intratumoral injection of CDNs produces a significant antitumor T cell immune response, prevents distal metastasis of lung cancer, generates immune memory, and causes complete tumor regression (Corrales et al., 2015). One of their CDNs was named ADU-S100. In 2019, Meric-Bernstam et al. reported that in comparison to cGAMP, ADU-S100 shows better metabolic stability and higher effectivity in activating STING (Meric-Bernstam et al., 2019). Currently, ADU-S100 is undergoing clinical trials as a STING agonist.

BMS-986301, a cyclodinucleotide derivative originally developed by IFM Therapeutics, was presented by Bristol Myers Squibb (BMS) at the Cancer ImmunoTherapy Society (SITC) in 2018. In May of 2019, BMS-986301 entered a phase 1 trial (Clinical Trials.gov ID: NCT03956680) to treat advanced solid tumors with monotherapy combined with immune checkpoint inhibitors (ICIs). However, the structure of BMS-986301 has not been fully determined (Ding et al., 2020).

In 2018, Harrington et al. reported that cyclic dinucleotide MK-1454 induced complete tumor regression through intratumoral administration and enhanced the efficacy of anti-PD-1 therapy in a homologous mouse tumor model (Harrington et al., 2018). MK-1454 has entered a clinical trial (NCT03010176) to treat advanced solid tumors (Harrington et al., 2018). These drugs have a high safety profile, and their maximum tolerated dose (MTD) has not been determined and should be further studied.

#### Nonnucleotide Agonists

##### Amide Compounds

Cyclic dinucleotide agonists are limited in their clinical application due to their high polarity and proteolytic tendency. In recent years, non-nucleotide derivatives have gained prominence due to their high specificity and effectiveness. In 2018, Ramanjulu et al. reported the synthesis of symmetrically related amide benzimidazole (ABZI) compound, which enhanced the binding and cellular function of STING (Ramanjulu et al., 2018). This formamide compound is anchored to Ser241 in the STING CTD region by hydrogen bonds. The pyrazole ring is located at the bottom of the binding pocket and connected to Ser162 by hydrogen bonds. In addition, two ABZI subunits on N1 benzimidazole bind to the pocket. These effects significantly enhance the binding affinity between STING and di-ABZI. In human peripheral blood mononuclear cells (h-PBMCs), di-ABZIs can induce IFN-β with EC_50_ of 130 nM, 400 times higher than cGAMP, without apparent toxicity. Furthermore, di-ABZI caused significant tumor volume regression by intravenous administration in a mouse model of colon tumors (Ramanjulu et al., 2018). In 2020, Xi et al. reported that amino benzimidazole derivatives - Compound 16g, 24b, and 24e, all STING agonists significantly increase the expression of IFN-β, CXCL10, and IL-6 and promote the phosphorylation of STING, TBK1, and IRF3 in h-PBMC and THP-1 cells (Xi et al., 2020). They also have significant antitumor effects when given intravenously in mice with colorectal tumors. Compounds 16g, 24b, and 24e in THP-1 cells showed high safety with the EC_50_ values of 1.24, 0.287, and 1.14 μM (Xi et al., 2020). Since then, N-(methylcarbamoyl)-2-{[5-(4-methylphenyl)-1,3,4-oxadiazol-2-yl]sulfanyl}-2-phenylacetamide (C11) (Gall et al., 2018) and 6-bromo-n -(naphthalen-1-yl)-benzo (d) (Stetson and Medzhitov, 2006; Ablasser et al., 2013) dioxole-5-carboxamide (BNBC) (Zhang et al., 2019) have been identified as h-STING agonists. In human fibroblasts (THF) and myeloid cell lines (MM6), C11 and BNBC can specifically activate STING-mediated transcription and translation of interferon and other antiviral genes, effectively blocking replication of multiple alphavirus types, including chikungunya fever, venezuelan equine encephalitis, mayaro virus. Moreover, the immune response is independent of MAVS and TRIF. However, C11 does not activate innate immune responses in mouse and THP-1 cells, and the specific mechanism is unclear.

##### Kitacinnamycins 8

The natural products of medicinal plants have been important resources for discovering novel drugs in recent decades (Newman and Cragg, 2016). In 2019, Shi et al. identified a new class of cinnamoyl-containing nonribosomal peptides (CCNPs) through the genomic collection and biosynthetic methods, named kitacinnamycins (Shi et al., 2019). Kitacinnamycins 8 increased poly (dA:dT) and cGAMP-induced IFN-β expression in a dose-dependent manner (Shi et al., 2019). However, the pharmacokinetics of kitacinnamycins 8 remain uncovered.

##### Flavonoids Compounds

In 2013, Kim et al. reported that 5,6-dimethylxanthenone-4-acetic acid (DMXAA) is a mouse STING agonist. In the mouse macrophage cell line Raw264.7 and L929 cells, DMXAA, similar to cyclic dinucleotide PAMPs and cyclic GMP-AMP, binds with m-STING to activate the TBK1-IRF3 pathway. In addition, DMXAA showed good activity in mouse solid tumors, causing tumor-specific vascular injury and other antitumor effects. However, its clinical trials failed, possibly because DMXAA does not bind to h-STING and lacks efficacy or mechanism-related toxicity in humans (Kim et al., 2013a). Unlike DMXAA, flavonoids α-Mangostin (Zhang et al., 2018) and G10 (Banerjee et al., 2020) were agonists of human STING. α-Mangostin and G10 bind to and stabilize the CTD region of STING in THP-1 cells and HEK293T (the human embryonic kidney cell line), respectively, and activate the STING-TBK1-IRF3 pathway. The EC_50_ values of G10 in STING R232 and H232 variants were 2.5 and 4.3 μM, respectively. In addition, α-Mangostin can repolarize human monocyte-derived M2 macrophages into the M1 phenotype, which has an antitumor function. However, α-Mangostin lacks *in vivo* biological activity and pharmacological properties, and G10 cannot activate all human STING, such as THP-1. In 2017, Liu identified a dispiro diketopiperzine (DSDP) compound as a h-STING agonist using a high-throughput cell-based screening method (Liu et al., 2017). DSDP induced STING-dependent cytokine responses in human foreskin fibroblasts (HFF) and h-PBMCs and effectively inhibited the replication of yellow fever virus, dengue virus, and Zika virus (Liu et al., 2017).

##### Other Compounds

In 2020, Pan et al. reported a non-nucleotide agonist, MSA-2, acting on STING (Pan et al., 2020). MSA-2 exists as an interconverting monomer or dimer, but only can it bind and activate STING in the dimer form. In mouse tumor models, MSA-2 was well tolerated by subcutaneous injection and oral administration. It can stimulate the secretion of IFN-β in tumors, induce tumor regression, have long-lasting antitumor immunity, and synergize with anti-PD-1 therapy. Moreover, in the acidic tumor microenvironment, the cellular efficacy of MSA-2 was enhanced with extracellular acidification (Pan et al., 2020). In 2020, Chin et al. reported another non-nucleotide STING agonist, SR-717 (Chin et al., 2020). SR-717 activates STING and induces “closure” of STING in a binding manner similar to that of cGAMP-STING. Through protein thermal transfer analysis, SR-717 can directly bind to recombinant STING and promote the cross-initiation of antigen and the activation of CD8^+^ T cells, natural killer cells, and dendritic cells. In addition, SR-717 can induce the expression of relevant immune genes, including programmed cell death ligand 1 (PD-L1), and show antitumor activity (Chin et al., 2020). MSA-2 and SR-717 are STING agonists suitable for clinical application because of their oral characteristics and simplified administration mode, and their pharmacological metabolism should be further studied.

## Indirect Modulation of the cGAS-STING Pathway

### Indirect Inhibition of cGAS by Targeting BAF

BAF is a self-integration disorder factor encoded by BANF1 and belongs to chromatin-binding protein, essential for nuclear membrane recombination in mitosis (Wang et al., 1996). BAF can dynamically bind to dsDNA, inhibit cGAS activity and suppress abnormal immune responses (Wang et al., 1998). Therefore, activating the cGAS-STING signaling pathway by inhibiting BAF may be an effective antitumor strategy (Zhao et al., 1998). Kim et al. found that a butanol lactone derivative, obtusilactone B, purified from spirea pernifolia, can inhibit BAF activity (Kim et al., 2013b). The specific binding of obtusilactone B to BAF inhibits vaccinia-associated kinase 1 (VRK1)-mediated BAF phosphorylation, leading to DNA nuclear membrane disintegration, thus inactivating BAF. In addition, Kim et al. isolated brazilin from legumes, which can inhibit BAF phosphorylation *in vitro* and *in vivo* by inhibiting VRK1, and disrupt BAF banding to DNA (Kim et al., 2015). Therefore, obtusilone B and brazilin can be candidates for the indirect regulation of cGAS-STING signaling. The above modulators are shown in Table 4.

**TABLE 4:** Indirect regulators targeting the cGAS-STING pathway.

Agents	Inhibition mechanism	Biological effect	Refs
Obtusilactone B, Brazilin	It inhibits dsDNA by inhibiting BAF	Indirect regulation of cGAS	Kim et al. (2013b); Kim et al. (2015)
Tucatinib	Inhibition of HER2 kinase activity	Indirect regulation of STING	Kulukian et al. (2020)
EGCG	Inhibits the enzyme activity of G3BP1	Indirect regulation of cGAS	Liu et al. (2019)
Compound C	Inhibit the accumulation of cGAMP	Indirect regulation of the cGAS-STING pathway	Lai et al. (2020)
Celastrol	Inhibit the activation of IRF3	Indirect regulation of the cGAS-STING pathway	Liu et al. (2020)
α,β−metADP/ATP,bzATP, ARL 67156	Inhibit the activation of ENPP1	Indirect regulation of the cGAMP	Lévesque et al. (2007); Lee et al. (2017)
(TiW11CoO40)8^−^, SR-8314, MV-626	Inhibit the activation of ENPP1	Indirect regulation of the cGAMP	Lee and Müller, (2017); Weston et al. (2019)

### Indirect Inhibition of STING by Targeting HER2

Studies have shown that tyrosine kinase receptor HER2 can effectively inhibit cGAS-STING signaling (Kroemer et al., 2015). Activated HER2 recruits the downstream protein kinase AKT1 and phosphorylates TBK1, thus blocking the formation of STING and TBK1 complex, and causing ubiquitination of TBK1 and ultimately weakening the STING signal (Wu et al., 2019). Therefore, inhibiting HER2 may effectively activate cGAS-STING-mediated signaling. Kulukian et al. reported a small molecule, tucatinib which could inhibit HER2 activity and blocks downstream signal transduction through MAPK and PI3K/AKT pathways (Kulukian et al., 2020). In addition, tucatinib was selectively cytotoxic to HER2-amplified breast cancer cells. Tucatinib has shown enhanced antitumor activity in combination with trastuzumab (therapeutic agents that target HER2 positive advanced metastatic tumors) or docetaxel (a newly developed taxoid anticancer agent), resulting in improved rates of partial and complete tumor regression (Kulukian et al., 2020).

### Indirect Inhibition of cGAS by Targeting G3BP1

In 2019, Liu et al. reported a novel cGAS-regulatory factor G3BP1 (GTPase activating protein SH3 domain-binding protein 1) (Liu et al., 2019). G3BP1 promotes the binding and activation of cGAS with DNA by changing the structure or oligomerization state of cGAS. Epigallocatechin gallate (EGCG), a polyphenol isolated from tea, is a known inhibitor of G3BP1 and specifically inhibits the binding of G3BP1 to cGAS and prevents the activation of cGAS, thereby blocking IFN-I production *in vivo* and *in vitro*. EGCG administration attenuated autologous DNA-induced autoinflammation in AGS mouse models and reduced interferon gene expression (Liu et al., 2019). Currently, EGCG is undergoing clinical trials with the potential to treat cGAS-dependent immune disorders.

### Modulation of the cGAS-STING Pathway by Compound C

Compound C is known to be a reversible inhibitor of AMPK and ALKs protein kinases. However, our group found that Compound C could inhibit the expression of IFN-β and related interferon stimulating factors (ISGs) by inhibiting the accumulation of cGAMP in the cytoplasm (Lai et al., 2020). Liquid chromatography-mass spectrometry (LC-MS) data showed that Compound C could inhibit the expression of type I interferon by decreasing the accumulation of cGAMP. It plays a modulator role in cGAS-STING-mediated DNA sensing pathway, but this effect is independent of AMPK protein activity. The IC_50_ of Compound C in L929 cells is 40 μM. In addition, Compound C can rescue the autoimmune phenotype of Trex1 gene deletion in mice (Lai et al., 2020), indicating that Compound C can inhibit the cGAS-STING pathway by acting on cGAMP, which will lay a foundation for further structural optimization of Compound C, and revealing the structure-activity relationship between small molecule compounds and cGAS or STING proteins, and for the design, synthesis and bioactivity studies of related new compounds.

### Indirect Inhibition of cGAS-STING Pathway by Targeting IRF3

Celastrol is a bioactive substance isolated from *Tripterygium wilfordii*. In 2020, our group found that celastrol could inhibit IRF3 activation *in vitro* and *in vivo*, thus effectively inhibits exogenous DNA-induced IFN-I response, with an IC_50_ value of 145.7 ± 23.6 nM (Liu et al., 2020). In addition, celastrol significantly rescued autoimmune phenotypes in *Trex1* ^*−/−*^ mice, including myocarditis and abnormal interferon response (Liu et al., 2020). Therefore, celastrol may be used to treat autoimmune and interferon-related diseases, but its specific targets need further clarification.

### Indirect Inhibition of cGAMP by Targeting ENPP1

Exonucleotide pyrophosphatase/phosphodiesterase I (ENPP1), as a key phosphodiesterase, catalyzes the hydrolysis of ATP or GTP to AMP or GMP, which affect the activity of STING by degrading cGAMP (Onyedibe et al., 2019). ENPP1-targeting inhibitors are expected to treat diseases associated with the cGAS-STING pathway. ENPP1 inhibitors can be divided into two classes. The first group are nucleotide inhibitors. They are structurally similar to natural ENPP1 substrates and competitively bind ENPP1 with ATP or GTP, such as α,β-metADP/ATP, 2-mesADP/ATP and bzATP, with IC_50_ values ranging from 13 to 32 μM (Lee et al., 2017). In addition, γ-S-α,β-metATP derivative, ARL 67156 and Diadenosine boranophosphate derivative are also nucleotide inhibitors of ENPP1, but their pharmacological activities need to be further determined (Lévesque et al., 2007). The second class of non-nucleotide inhibitors include polyoxometalates (TiW11CoO40)8−, suramin, heparin, etc. (TiW11CoO40)8− is the most effective ENPP1 inhibitor at present, and its K_i_ is 1.46 nM (Lee and Müller, 2017). In addition, SR-8314 and MV-626 could increase tumor infiltration of CD3^+^, CD4^+^, and CD8+T cells and showed significant antitumor activity (Weston et al., 2019).

## Clinical Studies of Regulatory Agents Targeting the cGAS-STING Pathway

Various modulators that target the cGAS-STING pathway have moved towards clinical trials (Table 5, data from https://www.clinicaltrials.gov/ October 13, 2021). Currently, two cGAS inhibitors are in clinical trials. In 2015, suramin combined with paclitaxel in treating stage IIIB-IV breast cancer (NCT00054028) proved effective. In 2019, aspirin was used in the clinical trial (NCT04132791) to prevent and treat cardiovascular diseases due to its ability to reduce the morning activity of platelets. A low-dose aspirin study is currently underway to prevent heart and vascular disease, colon and rectal cancer (NCT03603366). These clinical trials need to be followed up. It is believed that suramin and aspirin may be used as cGAS inhibitors to treat DNA-mediated immune diseases based on these pharmacological findings.

**TABLE 5:** Clinical trials of regulatory agents targeting cGAS-STING pathways.

Control agents	Control targets	Clinical trials	Disease
Aspirin	h-cGAS	Not applicable (NCT04132791) (NCT03603366) a total of 2,269 studies	Cardiovascular disease, cancer of the colon and rectum
Suramin	h-cGAS	Phase 1/2 (NCT00054028) a total of 21 studies	Stage IIIB-IV breast cancer
CXA-10	h-STING	Phase 2 (NCT03422510) a total of 11 studies	FSGS
ADU-S100	h-STING	Phase 2 (NCT03937141) phase 1 (NCT02675439) phase 1 (NCT03172936)	Head and neck cancer, advanced/metastatic solid tumors or lymphomas
MK-1454	h-STING	Phase 2 (NCT04220866) phase 1 (NCT03010176)	Neck squamous cell carcinoma, advanced/metastatic solid tumor, or lymphoma
BMS-986301	h-STING	Phase 1 (NCT03956680)	Advanced solid tumor
DMXAA (ASA404)	m-STING	Phase 1 (NCT00863733) phase 1 (NCT00856336) phase 1/2 (NCT00832494) a total of 18 studies	Solid tumors, DART, advanced non-small cell lung cancer
Tucatinib	HER2	Phase 1/2 (NCT03054363) phase 2 (NCT04579380) phase 2 (NCT03043313) phase 1/2 (NCT04430738) a total of 34 studies	Breast cancer, solid tumors, HER2+ colorectal cancer, HER2+ gastrointestinal cancers
EGCG	G3BP1	Phase 2 (NCT04553666) phase 0 (NCT02891538) phase 1 (NCT04177693) a total of 117 studies	Older cancer, colorectal cancer, pharmacokinetics, and hepatic safety

On the other hand, there are currently five STING modulators under clinical studies. From 2016 to 2019, ADU-S100 (NCT03937141, NCT02675439, NCT03172936), MK-1454 (NCT04220866, NCT03010176), and BMS-986301 (NCT03956680) were enrolled in clinical trials for the treatment of advanced/metastatic solid tumors or lymphomas. Compared with single-drug treatment, ADU-S100, MK-1454, or BMS-986301 combined with the ICIs therapy (Pembrolizumab/Ipilimumab/Nivolumab) to treat solid tumors and had shown good drug tolerance, has yet to reach maximum tolerated dose. Currently, CXA-10 has been used in 11 clinical trials. Among them, the clinical trial of oral CXA-10 in the treatment of primary focal segmental glomerulosclerosis has entered phase 2, but no clinical trial of CXA-10 as a STING inhibitor in the treatment of related immune diseases has been reported. In 2008, DMXAA was used as a STING agonist in clinical trial treating refractory tumors (DART). However, as it was an m-STING specific agonist, it did not react with h-STING, resulting in an unsatisfactory effect, and the experiment failed. Therefore, the structure of DMXAA needs to be further optimized. In addition, two indirect regulators targeting the cGAS-STING pathway have been tested clinically for pharmacologic metabolic research and cancer treatment.

In 2019, EGCG was studied in pharmacokinetics and liver safety pharmacology (NCT04177693). So far, 117 clinical trials related to EGCG have been enrolled.

By October 2021, tucatinib has been used in more than 30 clinical trials, particularly used for treating HER2^+^ breast cancer. For example, tucatinib (NCT03054363) is combined with palbociclib (a drug used to treat advanced breast cancer) and letrozole (aromatase inhibitor) used for treating hormone-receptor-positive and HER2-positive metastatic breast cancer patients. Treatment protocol of tucatinib with capecitabine and trastuzumab has been approved for treating patients with unresectable previously treated HER2^+^ breast cancer by US Food and Drug Administration on April 17, 2020.

## Summary and Prospect

In recent years, rapid progress has been made in clarifying the structure and mechanism of key proteins in the cGAS-STING pathway and in revealing the important role of this pathway in human autoimmune disease and cancer. Therefore, targeting the cGAS-STING signaling pathway to activate innate immunity and enhance the immune function provides great potential for cancer treatments. On the other hand, abnormal activation of the cGAS-STING pathway is the main cause of inflammation and autoimmune diseases. Therefore, the research and development of appropriate compounds, delivery pathways, and treatment regimens to suppress the cGAS-STING pathway will benefit patients with autoimmune and infectious diseases.

The crystal structures of several cGAS-STING pathway-related proteins have been analyzed, laying a foundation for the design of structure-based drugs. We now understand more clearly that binding or catalytic sites targeting cGAS and STING proteins and post-translational modifications can influence the enzyme activity and thus regulate immune responses. However, the high-resolution structures of some key protein complexes in the cGAS-STING pathway have not been resolved, such as the STING oligomer -TBK1 oligomer. It was found that the function of STING was strictly regulated by membrane transport, and retrograde membrane transport was crucial for silencing signaling pathways. This transportation defect is the basis of the pathogenesis of COPA syndrome, a single-gene autoinflammatory disease. The membrane transport of STING is co-mediated by COP-II and COP-I. Thus, using the regulatory agents to target membrane transport is likely to be a novel strategy for treating autoimmune diseases (Taguchi et al., 2021). However, the regulatory factors of STING transfer from the Golgi apparatus to the lysosome and the mechanisms of STING, NF-κB, and autophagy remain to be further studied. In addition, how post-translational modifications regulate the STING and other related enzyme activities, such as the relationship between palmitoylation of STING and oligomerization and activity, also remains unknown (Zhang et al., 2020). In recent years, cGAS has been closely related to the functions of histone and chromatin in the nucleus, and its interaction can affect the activity, but its structural basis and mechanism remain unclear (Kim et al., 2015).

On the other hand, agonists of the cGAS-STING pathway have potential value in the treatment of cancer. Some modified CDN analogs have entered clinical trials, but their clinical application may be hindered by their drug similarity, which needs to be treated in combination with ICIs, with collaborative administration. Small molecule non-CDN agonists provide a new strategy for systemic delivery, but clinical data have not been reported and need to be further verified. A potentially serious problem with agonist immunotherapy is the occurrence of “cytokine storms” (Ng et al., 2018). Continuous activation of immune signals can lead to excessive production of cytokines, causing severe toxicity or even death (Fu et al., 2020). Therefore, how much patients with autoimmune disease or cancer will benefit from cGAS-STING immunotherapy requires further investigation.

The pharmacodynamics optimization of cGAS-STING regulators and the rediscovery of natural drugs are important strategies for its immunopharmacology research. In addition, the compounds that indirectly regulate this pathway will also be a good focus for the study. Recently, inhibition of the cGAS-STING signaling pathway by nucleosomes (Boyer et al., 2020) and circRNAs (Xia et al., 2018) has been reported. Targeting the cGAS-STING pathway has promoted the vigorous development of immunotherapy. The combination of immunoregulatory agents and ICI_S_ therapy has become a hot spot in recent years. We anticipate that there will be more efficient and less toxic immune regulatory agents targeting cGAS-STING available in the future and applied for clinical practice to provide safer and more effective treatments for autoimmune disease and cancer.
